# Kawasaki disease complicated by macrophage activation syndrome treated with canakinumab

**DOI:** 10.70962/jhi.20250240

**Published:** 2026-04-09

**Authors:** Stejara A. Netea, Diana van Stijn, Mariken P. Gruppen, J. Merlijn van den Berg, Nico A. Blom, Irene M. Kuipers, Taco W. Kuijpers

**Affiliations:** 1 Department of Pediatric Immunology, Rheumatology and Infectious Diseases, Emma Children’s Hospital, Amsterdam University Medical Center, University of Amsterdam, Amsterdam, The Netherlands; 2 Department of Experimental Immunology, Amsterdam Institute for Infection and Immunity, Amsterdam University Medical Center, University of Amsterdam, Amsterdam, The Netherlands; 3 Pediatric Cardiology, Emma Children’s Hospital, Amsterdam University Medical Center, University of Amsterdam, Amsterdam, The Netherlands; 4 Department of Blood Cell Research, Sanquin Research Institute, University of Amsterdam, Amsterdam, The Netherlands

## Abstract

An infant presented with macrophage activation syndrome as part of Kawasaki disease, and was first treated with canakinumab, followed by methylprednisolone and IVIG. Despite early treatment, giant coronary artery aneurysms developed transiently, questioning the singular role of IL-1 in KD.

## Teaser

Netea et al. present an infant with macrophage activation syndrome as part of Kawasaki disease, who was first treated with canakinumab, followed by methylprednisolone and IVIG. Despite early treatment, giant coronary artery aneurysms developed transiently, questioning the singular role of IL-1 in KD.

Kawasaki disease (KD) is a pediatric hyperinflammatory syndrome with a risk of developing coronary artery aneurysms (CAAs) (1). KD patients with recrudescent or persistent fever after intravenous immunoglobulins (IVIG) infusion have an increased CAA risk. The optimal treatment in IVIG-resistant patients is uncertain (1). Previous findings, including genetic studies and experimental KD mouse models, suggest critical involvement of the interleukin-1 (IL-1) pathway (1, 2). In 2012, we reported for the first time the beneficial use of anakinra, an IL-1 receptor antagonist, in a child with severe relapsing KD, with normalization of giant CAAs within 6 mo (3). The beneficial use of anakinra was further supported by other clinical trials (1). In this context, we initiated the Kawakinumab study, a pilot proof-of-principle open-label study investigating the safety and efficacy of canakinumab in KD patients (NL68717.018.19). Canakinumab is a high-affinity human monoclonal anti-IL-1β antibody (IgG1/κ). Its longer half-life of 23–26 days enables administration of a single dose instead of daily infusions. Unlike anakinra, a recombinant IL-1-receptor antagonist, blocking both IL-1α and IL-1β, canakinumab only neutralizes human IL-1β. Evidence from elevated serum IL-1β levels in KD patients, genetic studies, reduced IL-1β release after treatment, and mouse models suggests that IL-1β is more likely a key driver of KD pathogenesis than IL-1α (1, 2). Furthermore, canakinumab has demonstrated efficacy in several IL-1β-driven inflammatory diseases (e.g., cryopyrin-associated periodic syndromes, systemic juvenile idiopathic arthritis/macrophage activation syndrome [MAS], and periodic fever syndromes). In this context, we initiated the Kawakinumab study, incorporating careful clinical and laboratory monitoring and ensuring a safe window to administer IVIG within 10 days of symptom onset, minimizing CAA risk.

Here, we report the first treatment-naive KD patient treated with a single high-dose infusion of canakinumab in the context of concurrent MAS.

**Figure 1.**
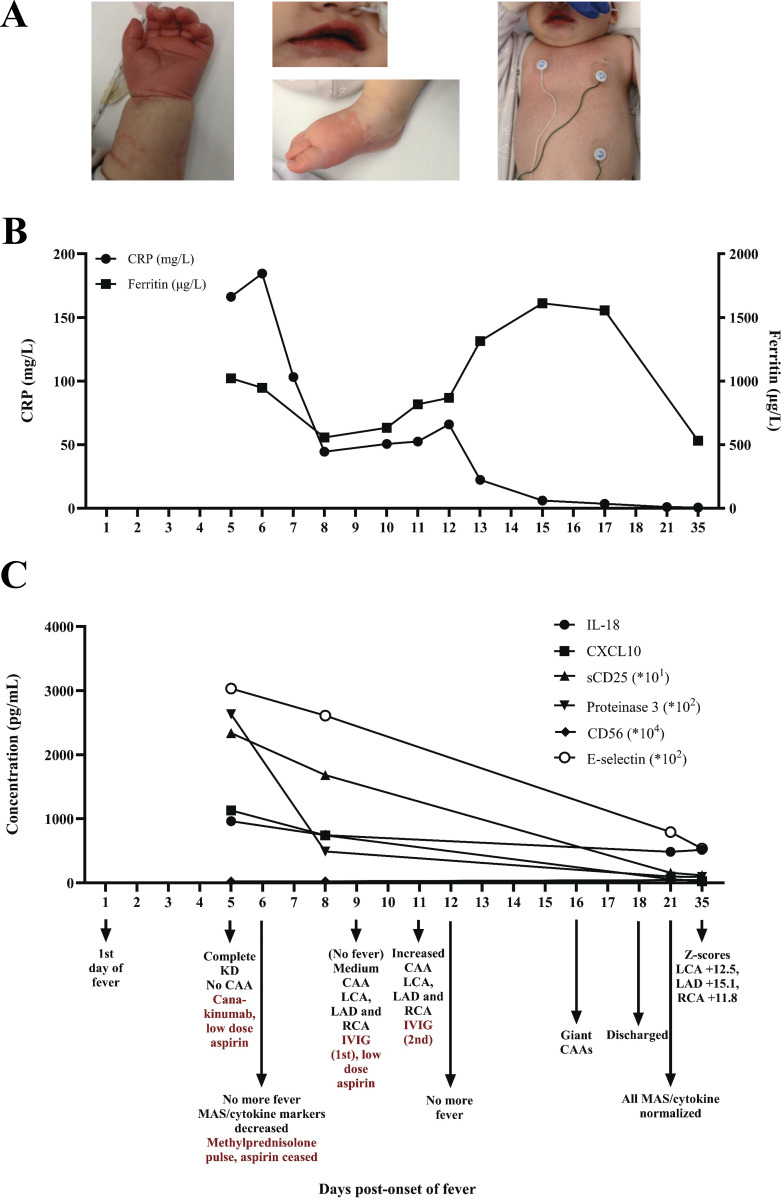
**Key clinical features of the case, CRP and ferritin levels, and inflammatory protein values over time.** **(A–C)** Clinical features, including erythema of the extremities, red and chapped lips, and rash (A), visual summary (B) of KD case presented, and (C) serial values of inflammatory proteins measured by Luminex.

A 5-mo-old female patient was admitted for complete KD with 5 days of fever (Fig. 1). Upon admission, she had hepatosplenomegaly, a raised C-reactive protein (CRP, 166.3 mg/L), hypoalbuminemia (17 g/L), anemia (Hb 5.1 mmol/L), thrombocytopenia (50 × 10^9^/L), raised triglycerides (2.9 mmol/L [N < 1.5 mmol/L]), ferritin (1,022 µg/ml [N < 250 µg/ml]), IL-18 (963 pg/ml [N < 175 pg/ml), N-terminal pro-B-type natriuretic peptide (15,592 ng/L [N ≤ 86 ng/L]), and normal fibrinogen (1.9 g/L). Additional inflammatory proteins (IL-18, C-X-C motif chemokine ligand 10 [CXCL10], soluble cluster of differentiation 25 [sCD25], cluster of differentiation 163, galectin-3-binding protein, C-X-C motif chemokine ligand 1, lipocalin-2, proteinase 3, cluster of differentiation 56, E-selectin, angiopoietin-2, and tissue inhibitor of metalloproteinases-1) were quantified using Luminex. Various of these, including soluble sCD25, CXCL10, proteinase 3, E-selectin, and angiopoietin-2, were raised (Fig. 1 C). No CAAs or cardiac dysfunction were seen. She was treated with canakinumab (single dose, 6 mg/kg) and subsequently suspected of MAS. Aspirin was initiated in a low dose of 4 mg/kg/day (normally started 30–50 mg/kg/day and reduced to 3–5 mg/kg/day once fever has subsided for 24 h [1]) due to thrombocytopenia with increased risk of bleeding and no evidence of giant CAA. Within several hours, the fever, irritability, redness of her lips, and rash had subsided. However, laboratory results the next morning indicated persisting inflammation (CRP 184.6 mg/L, Hb 4.8 mmol/L, thrombocytes 19 × 10^9^/L, ferritin 947 µg/ml, and triglycerides 2.9 mmol/L), and methylprednisolone (30 mg/kg/day, 3 days) was started, ∼8 h following the canakinumab infusion. Aspirin was discontinued given the further decrease in thrombocytes. Despite clinical improvement (no fever or KD stigmata, improving laboratory parameters [also those measured by Luminex, Fig. 1 C]), the patient developed CAAs on day 9 after onset (z-scores left coronary artery [LCA] +5.2, left anterior descending artery [LAD] +5.5, and right coronary artery [RCA] +7.1). IVIG (single infusion of 2 g/kg) and aspirin (4 mg/kg/day, in the presence of normalizing platelet counts) were initiated, and oral prednisolone (1 mg/kg/day) continued. Because of further coronary dilatation (z-scores LCA +8.0, LAD +6.4, and RCA +8.1), a second IVIG infusion (2 g/kg) was given on day 11, and therapeutic low-molecular weight heparin was started. Within 4 days, the CAAs progressed to giant CAAs (z-scores LCA +12.5, LAD +15.1, and RCA +11.8), but the patient improved clinically and went home after 10 days of admission. During the first 6 wk of follow-up, the patient did not develop fever, and the coronary artery diameters remained stable. The patient was closely monitored (1 year since the acute disease episode), and the maximum CAA z-score of the LCA has regressed to +5.3 and of the RCA to +2.7. Inflammatory markers measured by Luminex normalized within 3 wk after the disease.

In this KD case with concomitant signs of MAS, canakinumab treatment suppressed several major clinical manifestations, but the development of CAAs could not be prevented. Despite evidence implicating IL-1β as a key mediator in KD pathogenesis (1, 2, 4), the current case suggests that IL-1β blockade alone may not be sufficient to prevent all disease complications.

The early presentation of MAS enabled timely initiation of biological therapy and IVIG <10 days onset. Although MAS in KD generally responds well to treatments such as corticosteroids and monoclonal antibodies, CAAs still developed in this case despite adequate therapy with a relatively high dose of 6 mg/kg. Various aspects of the current case are therefore important to take into consideration for future treatment strategies in complex cases of KD.

First, a likely explanation for the development of CAAs is that our patient had several risk factors. She had a very young age at onset (<6 mo) and presence of MAS, which are associated with a markedly increased CAA risk ∼35–50% (1).

Second, the limited response to canakinumab may, at least in part, be related to its selective blockade of IL-1β without targeting IL-1α. However, consistent with our rationale underlying the initiation of this study, we hypothesize IL-1β is a more likely key contributor in KD pathogenesis than IL-1α (2).

Third, the timing and pharmacologic differences between IL-1 inhibitors may affect the limited clinical response in KD, compared to MAS. If IL-1 blockers are given before IVIG has neutralized unidentified pathogenic triggers involved in this disease to temper the acute inflammation, their clinical effect may be less beneficial. More importantly, the short half-life of anakinra (∼4–6 h) enables rapid onset/offset, close titration to clinical response, and prompt discontinuation in case of inefficacy or adverse events, albeit at the cost of requiring painful daily injections. In contrast, canakinumab has a much longer half-life (∼23–26 days), resulting in prolonged IL-1β blockade after a single administration, albeit without blocking IL-1α. These differences may account for the differences with previous reports supporting the benefit of anakinra in both MAS and KD.

Fourth, the presented case did not receive primary corticosteroid treatment in combination with canakinumab. Although current guidelines state that adding primary corticosteroids to IVIG and aspirin may be considered in high-risk acute KD patients, this approach has not been consistently implemented in local protocols due to conflicting trial results and the lack of evidence in non-Asian populations (1). Moreover, we did initiate corticosteroids ∼8 h after starting canakinumab, as soon as it became clear that canakinumab alone had not fully controlled the MAS instantly.

Finally, our case supports the view that KD represents a clinical syndrome driven by different inflammatory drivers, potentially requiring tailored therapeutic strategies, which at the moment are not yet well-defined. The biomarkers in the current patient (Fig. 1 C) are most consistent with IFN-γ–associated immune activation during the acute phase. Specifically, CXCL10, an IFN-γ–inducible chemokine, and sCD25, a marker of T cell activation, were markedly elevated early after fever onset. IL-18 was also elevated, indicating concurrent activation of the IL-1 family axis. This supports an inflammatory signature similar to MAS, marked by increased levels of IL-18 and resulting in the induction and release of CXCL10. Interestingly, though, TNF-axis-related biomarkers, including IL-6 and IL-17A, were very low, similar to controls. As yet, it is still unknown if these profiles can be directly related to therapeutic effects. Nevertheless, emerging data from large cohort studies indicate that TNF-α blockade, particularly with infliximab, may hold significant promise for high-risk or refractory cases following IVIG infusion as mainstay treatment. It has been demonstrated that infliximab effectively reduces fever duration, and the need for additional therapy, possibly even lowering the risk of coronary complications (1, 5). Whether TNF-α blockade is as effective without prior IVIG remains unknown. Nonetheless, in light of these promising results and the findings of our presented case, we have decided to halt the Kawakinumab trial (NL68717.018.19).

We conclude that in search of novel and safe treatment approaches, complicated therapy-resistant KD remains a challenging condition to treat adequately. While informative, animal models are of limited value to guide our treatment decisions in KD, leaving us with detailed case-by-case trial-and-error reports. Although we did observe good response of canakinumab in a KD case with recurrent fever following initial IVIG (data not shown), the current case highlights that primary IL-1β blockade with canakinumab is not sufficient to prevent disease progression in this single high-risk infant and raises the hypothesis that IL-1–targeted strategies may not be universally effective in complex or therapy-resistant KD. Further studies are needed to better define which patients may benefit from IL-1 blockade and to clarify the exact role of the different IL-1 inhibitors, including anakinra.

## Data Availability

The original study data are presented in the article. The corresponding author can be contacted for further inquiries.
